# Trends in the prevalence of antenatal and postnatal depression in Bangladesh: A systematic review and meta-analysis

**DOI:** 10.1016/j.heliyon.2025.e41955

**Published:** 2025-01-14

**Authors:** Mohammad Injamul Hoq, Md Mohotasin Hossain, Mohammad Aktar Sayeed, Md Jakaria

**Affiliations:** a Department of Pharmacy, International Islamic University Chittagong, Kumira, Chittagong, 4318, Bangladesh; b Department of Public Health, University of Creative Technology Chittagong, Chittagong, 4212, Bangladesh; c The Florey Institute, The University of Melbourne, Parkville, VIC, 3052, Australia; d School of Health Sciences & Purdue Institute for Integrative Neurosciences, Purdue University, West Lafayette, IN, 47907, USA

**Keywords:** Maternal depression, Antenatal depression, Postnatal depression, Prevalence, Bangladesh

## Abstract

**Background:**

Maternal depression negatively impacts the health of both mothers and their children. Although several studies have reported on the prevalence of antenatal depression (AND) and postnatal depression (PND) in Bangladesh, reliable estimates based on meta-analysis have yet to be established. This study aims to determine the prevalence of “AND” and “PND” among Bangladeshi mothers, as well as the prevalence of “PND” during various phases of a child's development, while also identifying the associated factors for both “AND” and “PND”.

**Methods:**

We conducted a systematic search in PubMed, Scopus, Cochrane, and a national database called Bangla Jol for studies published from the year 2000 until December 31, 2020. From 163 screened studies, eighteen eligible studies on the prevalence of “AND” and “PND” were included for meta-analysis. A random-effects model was used for this analysis. We also performed subgroup analyses considering “PND” at different stages, study quality, and prevalence based on the decade of publication.

**Results:**

The pooled prevalence rates were found to be 19.5 % for “AND” (95 % CI: 7.7 %–31.28 %, I^2^: 98.09 %) and 27.75 % for “PND” (95 % CI: 22.38 %–33.16 %, I^2^: 97.67 %). In the 2000s, the pooled prevalence was 22.78 % (95 % CI: 17.82 %–27.73 %, I^2^: 96.65 %). However, there was a significant increase in the prevalence of “PND” in the 2010s, which reached 36.00 % (95 % CI: 23.94 %–48.06 %, I^2^: 95.76 %). The pooled prevalence at an early stage of the child's development was 17.12 %; during exclusive breastfeeding, it was 25.73 %, and during complementary feeding, it peaked at 48.11 %. Factors associated with maternal depression included unplanned pregnancies, various forms of intimate partner violence (including physical, emotional, and sexual violence), a preference for male children, and strained relationships with husbands and mothers-in-law.

**Conclusion:**

A rising trend in the prevalence of maternal depression has been observed in Bangladesh. Health policymakers need to prioritize addressing maternal depression. The data indicates that the prevalence of postpartum depression was higher in the 2010s compared to the previous decade. It is crucial to raise awareness among mothers about the importance of screening for depression during the perinatal period, and to integrate such screenings into family planning and mental health services.

## 1 Introduction

The emotional, physical, and behavioral changes during pregnancy and after childbirth that last for more than two weeks among women are termed antenatal depression (AND) and postnatal depression (PND), respectively. Due to the temporary alteration of hormonal state, women are more susceptible to developing depression in pregnancy compared to other periods of their life [1]. Depression during the perinatal period is often ignored even though significant physical and psychological changes occur among women during and after pregnancy [2]. Only 25 % of women who experienced moderate to severe depression sought care for their emotional healing [3].

A meta-analysis report stated that “AND” prevalence ranges between 15% and 65 % and “PND” was almost between 13 % and 31.1 % within a year of childbirth worldwide [[4], [5], [6]]. The “AND” and “PND” prevalences were 15.6 % and 16.84 %, respectively, in developing regions like Africa [7,8]. A global study demonstrated high-income countries had “AND” prevalence ranges from 7% to 15 % whereas low- and middle-income countries had a prevalence of 19–25 % [9]. In the Asian region, the prevalence of depression was 63.3 % among postpartum women [10]. In the Southeast Asian region, the prevalence varies from 4.4 % to 57.7 % [11], which indicates a significantly higher prevalence of “PND” in this region than the world prevalence (17.2%–17.7 %) [12,13]. A population-based study in Sri Lanka reported a “PND” prevalence of 9.4 % [14]. India and Bangladesh reported a depression prevalence of 11–16 % and 18–35 % after delivery, respectively [[15], [16], [17]], which indicates a higher magnitude of “PND” in Bangladesh among Southeast Asian countries.

Several factors reported being associated with depression in mothers, including poor health status, previous history of depression or anxiety, poor financial situations, lack of social support, low self-esteem, and domestic violence during pregnancy [18,19]. Growing evidence suggests that depression during the maternal period could affect productivity, quality of life, and child development [20]. Detrimental effects have been observed both in the mother's and child's health due to maternal depression; chronic recurrent depression among mothers, the child may face cognitive, behavioral, and emotional problems at their later stage of life along with a tendency of not practicing exclusive breastfeeding by mothers leads to child malnutrition, extreme diarrhea and may suffer from other infectious illness reported by several studies in low-middle income countries [[21], [22], [23], [24]].

World Health Organization's 2022 guidelines on maternal and newborn care provide an updated framework for screening and the management of maternal depression that reflects a global consensus on the importance of reducing maternal depression through early interventions [25]. On the other hand, the biopsychosocial model offers a holistic approach to understanding maternal mental health. However, there is a lack of knowledge on depression knowledge is scarce about the social, physical, and psychological impacts of depression in low- and middle-income countries [26]. Therefore, a mother's mental health during pregnancy or after childbirth is a crucial research concern among public health issues in developing countries like Bangladesh.

Maternal depression is getting attention in the broader framework of mental health policy according to the National Mental Health Act 2018 and National Mental Health Strategic Plan 2020–2030 in Bangladesh. However, public awareness, training community health workers to manage maternal depression, and routine screening of depression among pregnant and postpartum women are still pressing concerns [27,28].

Although there is a vast difference in prevalence rates across different studies with methodological weakness in measuring depression among mothers, there has been no systematic review and meta-analysis found in Bangladesh. Consequently, reliable estimates of maternal depression are yet to be established. This study determined the current prevalence of “AND” and “PND” among Bangladeshi mothers and “PND” prevalence in different stages of the postnatal period. We also determined the associated factors with “AND” and “PND” in Bangladesh. The study's findings on the pooled prevalence and associated factors of perinatal depression will be helpful for health policymakers to reconsider the issue and pay attention to establishing preventive strategies to build awareness of social destigmatization on “AND” and “PND” and further research.

## 2 Materials & methods

### 2.1 Study protocol

The protocol of the study was registered in PROSPERO (reg: CRD42021211538) [29].

### 2.2 Search strategy

PubMed, Scopus, Cochrane, and BanglaJOL were systematically searched. The search terms were divided into area, methodology, and outcome categories. 1. Area: Bangladesh; 2. Methodology: cross-sectional study, cohort study; 3. Outcome: “antenatal depression”, “postnatal depression”, “prenatal depression,” “perinatal depression,” “antepartum depression,” and “postpartum depression.” The “OR” Boolean operator was used for similar search terms, and the “AND” operator combined search terms across the categories. Only studies conducted on humans and published in English before 2020 were searched comprehensively for this review and meta-analysis. Search strategy has been included as a supplementary table in the supplementary files.

### 2.3 Inclusion & exclusion criteria

Studies that either had prevalence data on “AND” or “PND” from Bangladeshi women with cohort and cross-sectional designs were included. The studies published in English till December 31, 2020, were considered for inclusion. Studies dealing with samples from outside of Bangladesh and review studies on “AND” or “PND” were excluded from the meta-analysis. Case-series, case report study designs were excluded as they do not provide sufficient evidence for our research question. The qualitative studies were excluded from this review to maintain the consistency of quantitative measurement of included studies. As resources were unavailable for translation, studies published in other languages except English were excluded from this review. However, the majority of the articles were available in English. We acknowledge the limitation that poses a limitation to the generalizability of our findings.

### 2.4 Study selection

**Fig. 1:**
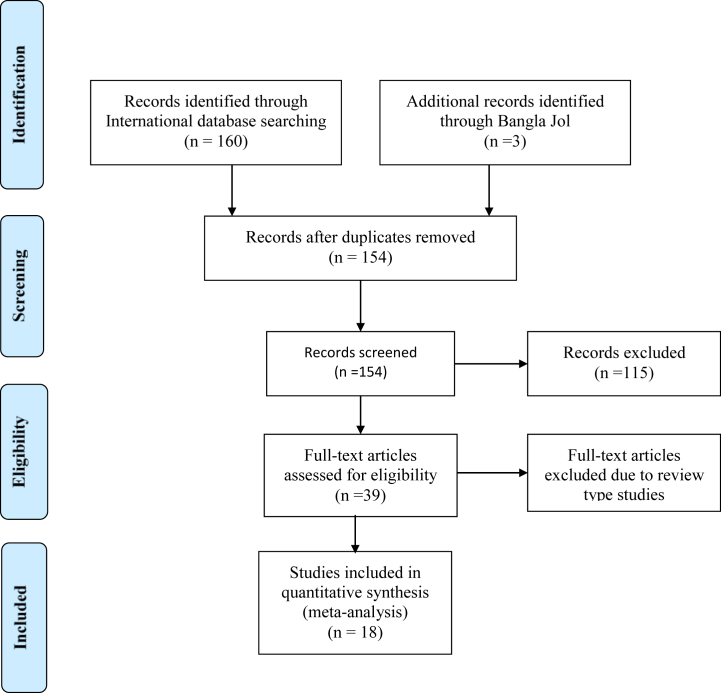
A Prisma Flow diagram to show the selection of the study process.

Two independent investigators, MMH and MIH, reviewed the eligible articles. In cases of disagreement, a third investigator (MJ) was consulted, and consensus was reached through discussion. The titles and abstracts were screened first, and articles were selected based on the inclusion criteria. A total of 163 studies were screened based on our search strategy. Nine studies were excluded for duplicity. After duplicate removals of these 154 studies, 115 had no prevalence data. Of the eligible 39 full-text studies, 21 were excluded from the qualitative or review-type study design. Finally, 18 original studies were included in the meta-analysis, which reported prevalence data either on “AND,” “PND” or both. Fig. 1 illustrates the study selection procedure.

### 2.5 Data extraction

Regarding data extraction, two reviewers independently extracted data and cross-checked it for consistency. If discrepancies were observed, a third investigator was consulted to resolve the issue through discussion and consensus. Data were extracted from selected articles using inclusion and

exclusion criteria. The following information was extracted from full-text reviewed articles: (A) author, publication year; (B) Title; (C) Study design; (D) Study location; (E) Sampling strategy; (F) Diagnostic criteria; (G) Sample size; (H) Antenatal and postnatal depression prevalence and prevalence by strata like depression during first three months after birth (termed as immediate phase), at first six months of birth (exclusive phase), depression after six months (complementary phase) and data collected before 2010 versus after 2010; (I) Outcome assessment (Objective or subjective); and (J) Risk factors or associated factors identified in each study.

### 2.6 Quality assessment

Three distinct characteristics were chosen to determine the quality of the reviewed studies: 1. Whether the sampling strategies of the studies (Random = 1, Non-random = 0). 2. Had adequate sample size or not (500 or more = 1, less than 500 = 0). 3. Whether disease outcome assessment by studies (Objective = 1, Subjective = 0). With this scoring, a study can achieve a maximum of 3 points and a minimum of 0 points. The studies that achieved 3 points were considered high quality, and those that achieved 2 or fewer points were labeled low quality. This categorization was adapted from an earlier meta-analysis conducted in Bangladesh [30].

Along with the quality assessment of reviewed studies, the systematic review was evaluated by the AMSTAR checklist shown in Table 1. The quality of the systematic review was assessed using the AMSTAR-2 checklist.

**Table 1:** AMSTAR-2 checklist for systematic reviews.

Domain	Response Options
**1. Did the research questions and inclusion criteria for the review include the components of PICO?**	**Yes**
**2. Was the review protocol registered before the conduct of the review (e.g., in PROSPERO)?**	**Yes**
**3. Did the review authors explain their selection of study designs for inclusion?**	**Yes**
**4. Did the review authors use a comprehensive literature search strategy?**	**Yes**
**5. Did the review authors perform study selection in duplicate?**	**Yes**
**6. Did the review authors perform data extraction in duplicate?**	**Yes**
**7. Did the review authors provide a list of excluded studies and justify the exclusions?**	**Yes**
**8. Did the review authors describe the included studies in adequate detail?**	**Yes**
**9. Did the review authors use a satisfactory technique for assessing the risk of bias (RoB) in individual studies?**	**Partial Yes**
**10. Did the review authors report on the sources of funding for the studies included in the review?**	**Yes**
**11. If meta-analysis was performed, did the review authors use appropriate methods for statistical combination of results?**	**Yes**
**12. If meta-analysis was performed, did the review authors assess the potential impact of risk of bias in individual studies on the results of the meta-analysis or other evidence synthesis?**	**Yes**
**13. Did the review authors account for RoB in primary studies when interpreting/discussing the results of the review?**	**Yes**
**14. Did the review authors provide a satisfactory explanation for, and discussion of, any heterogeneity observed in the results of the review?**	**Yes**
**15. If they performed quantitative synthesis, did the review authors carry out an adequate investigation of publication bias (small study bias) and discuss its likely impact on the results?**	**Yes**
**16. Did the review authors report any potential sources of conflict of interest, including any funding they received for conducting the review?**	**Yes**

The review adhered to most critical items, including comprehensive search strategies, duplicate study selection and data extraction, and assessment of the methodological quality of included studies. However, the review has addressed all critical aspects of systematic review methodology. This review would be classified as high quality, and it provides an accurate and comprehensive summary of the available studies.

### 2.7 Analysis plan

The methodology established by Neyeloff et al., 2012 was followed for the meta-analysis [31]. In the subgroup analysis, different stages of the postnatal period, quality-based study category (only “PND”), and decade-based “PND” prevalence were considered for calculation.

The three stages in the postnatal period had been epitomised as mother depression at the child's early stage, depression at the exclusive stage, and depression at the complementary stage. Later, two terms were adapted from exclusive breastfeeding and complementary feeding age of infants. The studies that achieved 3 points were considered high quality, and studies that achieved 2 or fewer points were labeled as low quality. In addition, the pooled prevalence of “PND” during the first and second decades of the 21st century (data collected before 2010 versus after 2010).

### 2.8 Data processing and analysis

After collecting data, it was checked and rechecked to ensure consistency. Data curation was done before data analysis. The data were verified, edited for consistency, compiled, tabulated, and analyzed. The calculated variables for each study were. 1. Standard error of the prevalence; 2. Variance; 3. Study weight; 4. Study weight × outcome estimate or prevalence; 5. Study weight × (outcome estimate)^2^; 6. (Weight)^2^. Furthermore, these variables were used to estimate Q (heterogeneity measurement among the studies) and I^2^ (Variability among studies). I^2^ value measures the degree of inconsistency across studies. 25 % and lower is considered low heterogeneity, and 75 % or more is regarded as high heterogeneity [32]. Based on Q and I^2^ values (high heterogeneity), the authors selected a random effects model to estimate pooled prevalence with a 95 % confidence interval of “AND” and “PND.” Publication bias was assessed using a funnel plot and Egger's test. The data were analyzed in Microsoft Excel ‘13, and R software, version 4.4.2. The analyzed data was portrayed by using R software, version 4.4.2 and Graph Pad prism, version 8.4.2.

## 3 Results

### 3.1 General characteristics of reviewed articles

Dhaka division was the study site for eleven studies. Out of eleven, seven studies choose Matlab as their study place, a rural area in Bangladesh. Four studies were conducted in the Mymensingh district. From the remaining three, one study was conducted at Chandpur in the Chittagong division, one at Ullapara, Sirajganj, under the Rajshahi division, and the other one at Gaibandha in the Rangpur district. A total of thirteen studies had cohort study design, and five were cross-sectional. Most of the studies recruited participants from rural areas and used a random sampling technique. Only two studies collected data from hospital or facility centers [33,34] and one from the slums of an urban area [35]. Of the 18 original studies, three included prevalence data on AND, and 16 had PND prevalence. Among them, one study shared prevalence for “AND” and “PND".

A maximum number of studies used the Edinburgh Postnatal Depression Scale (EPDS) or its Bangla version (EPDS-B) for measuring depression among mothers during pregnancy and after childbirth. However, two studies used the Center for Epidemiologic Studies Depression Scale (CESD), one administered the Patient Health Questionnaire (PHQ-9), and one used the Self-Reporting Questionnaire for measuring depression in pre-birth and post-birth period among mothers.

Studies defined postnatal depression as depressive symptoms after the child's birth. Studies followed mothers between 1 and 104 weeks after a child's birth. Among the “PND” reported studies, seven articles estimated depression among mothers at a very early stage of childbirth, which was within the first three months of childbirth. Eight studies reported depression at exclusive stages, and 4 studies collected data for measuring the depression status of mothers at the complementary stage of infants. Four studies outlined “PND” prevalence among mothers at two stages, and one study reported both “AND” and “PND” prevalence [36]. For instance, pregnant mothers were recruited during their third trimester to assess “AND” prevalence in the three studies.

Overall, twelve studies collected data before 2010, and six were published between 2010 and 2020. Based on quality assessment criteria, 11 studies were low quality, and 7 were high quality. The general characteristics of the included studies are demonstrated in Table 2.

**Table 2:** Antenatal depression (AND) and Postnatal depression (PND) prevalence in Bangladesh: A summary of epidemiological studies published in Bangladesh.

Author, Year	Study area	Study type	Sample size	Prevalence	Scales	Questionnaire period
Nasreen et al., 2010 [38]	Mymensingh district	Cohort study	720	Antenatal depression: 18 %	EPDS and STAI	Third trimester
Gausia et al., 2009 [37]	Matlab, Dhaka	Cohort study	361	Antenatal depression: 33 %	EPDS-B	34–35 weeks of pregnancy
Edhborg et al., 2011 [41]	Rural sub-districts of Mymensingh district	Cohort study	720	Postnatal depression: 11 %	EPDS and STAI	2–3 months postpartum
Azad et al., 2019 [35]	Urban slums (Gulshan, Mohakhali, Mohammadpur) from Dhaka city	Cross-sectional study	376	Postnatal depression: 39.4 %	EPDS	First 12 months following the childbirth.
Sharmin et al., 2019 [34]	Dhaka city	Cohort study	400	Postnatal depression: 25.7 %	EPDS	Followed up to 6–8 months
Parveen et al., 2017 [33]	Dhaka (BSMMU)	Cross-sectional study	145	Postnatal depression: 12.4 %	EPDS	day 5–7 following cesarean section
Edhborg et al., 2020 [40]	Mymensingh district	longitudinal study	656	Postnatal depression 31.6 %	EPDS	At 6–8 months postpartum
Surkan et al., 2018 [36]	Gaibandha and Rangpur Districts (rural)	Nested Cohort Study	Ante natal: 14,629 Postnatal: 31,422	Ante natal depression: 8.2 % Postnatal depressio:12.6 %	PHQ-9 and CES-D	Third trimester 6 months postpartum
Gausia et al., 2009 [16]	Matlab, Dhaka	Cohort study	346	Postnatal depression: 22 %	EPDS-B	6–8 weeks postpartum
Black et al., 2009 [39]	Matlab, Dhaka	Nested Cohort study	222	Postnatal depression: 52.7 %	CES-D	At 6 months and at 12 months
Kabir et al., 2014 [49]	Mymensingh district	Cross-sectional study	660	Postnatal depression: 32 %	EPDS	6–8 months postpartum
Hamadani et al., 2012 [45]	Matlab	Cohort study	6 weeks:512 6 months: 503	6 weeks postnatal depression: 17 % 6 months postnatal depression: 11 %	EPDS	6 weeks and 6 months postpartum
Gausia et al., 2012 [44]	Matlab Random	Cohort study	546	Postnatal depression: 15.4 %	EPDS	6 weeks of postpartum
Islam et al., 2017 [47]	Chandpur district	Cross-sectional	426	35.2 %	EPDS	6 months postpartum
Jiang et al., 2017 [48]	Dhaka, Mirpur	Cohort study	78 weeks: 205 104 weeks: 422	78 weeks: 55.1 % 104 weeks: 48.2 %	EPDS	78 weeks–104 weeks
Gausia et al., 2011 [43]	Matlab	Perspective community based (follow up)	476	6 weeks postpartum: 23.7 % 6 months postpartum: 12 %	EPDS-B	6 weeks-6 months postpartum
Gausia et al., 2010 [42]	Matlab, Dhaka	Cohort study	320	Postnatal depression:20 %	EPDS	6–8 weeks postpartum
Hossain et al., 2020 [46]	Ullapara, sub district of (rural)	Cross sectional study	591	Postnatal depression: 51.7 %	SRQ-20	6–16 months postpartum

### 3.2 “AND” prevalence among Bangladeshi women

**Fig. 2:**
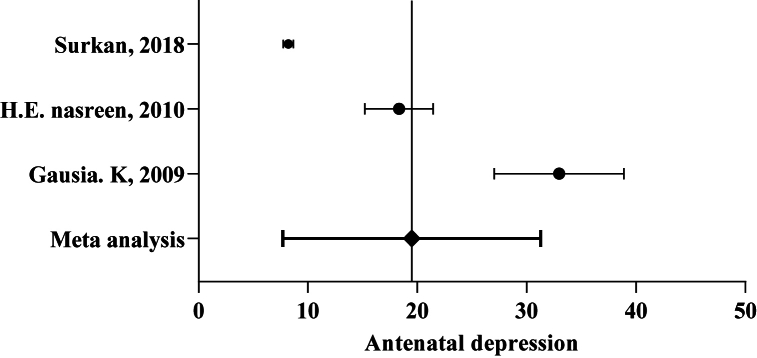
Meta-analysis of antenatal depression prevalence among Bangladeshi women (n = 3 studies; year = 2009–2018).

A total of 15,710 participants were capitalized from three studies [[36], [37], [38]] for measuring the pooled prevalence of “AND.” The overall prevalence of AND varied across studies from 8.2 % to 32.9 % (median: 18.33 %). The pooled “AND” prevalence was 19.5 % (95 % CI: 7.7 % ⎯ 31.28 %, I^2^:98.09 %) (Fig. 2).

### 3.3 “PND” prevalence among Bangladeshi women

**Fig. 3:**
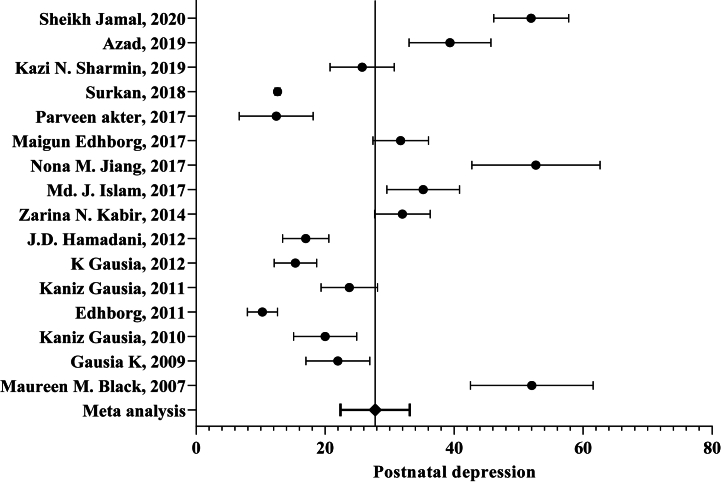
Meta-analysis of postnatal depression prevalence among Bangladeshi women (n = 16; year = 2007–2020).

We exploited a total of 38,023 participants from 16 studies [16,[33], [34], [35], [36],[39], [40], [41], [42], [43], [44], [45], [46], [47], [48], [49]] On “PND” prevalence for pooled prevalence. The overall prevalence of “PND” varied from 10.27 % to 52.68 %. The pooled prevalence was 27.75 % (95 % CI: 22.38 % ⎯ 33.16 %%, I^2^:97.67 %) (Fig. 3).

### 3.4 Prevalence of “PND” during different stages of the postnatal period

**Fig. 4:**
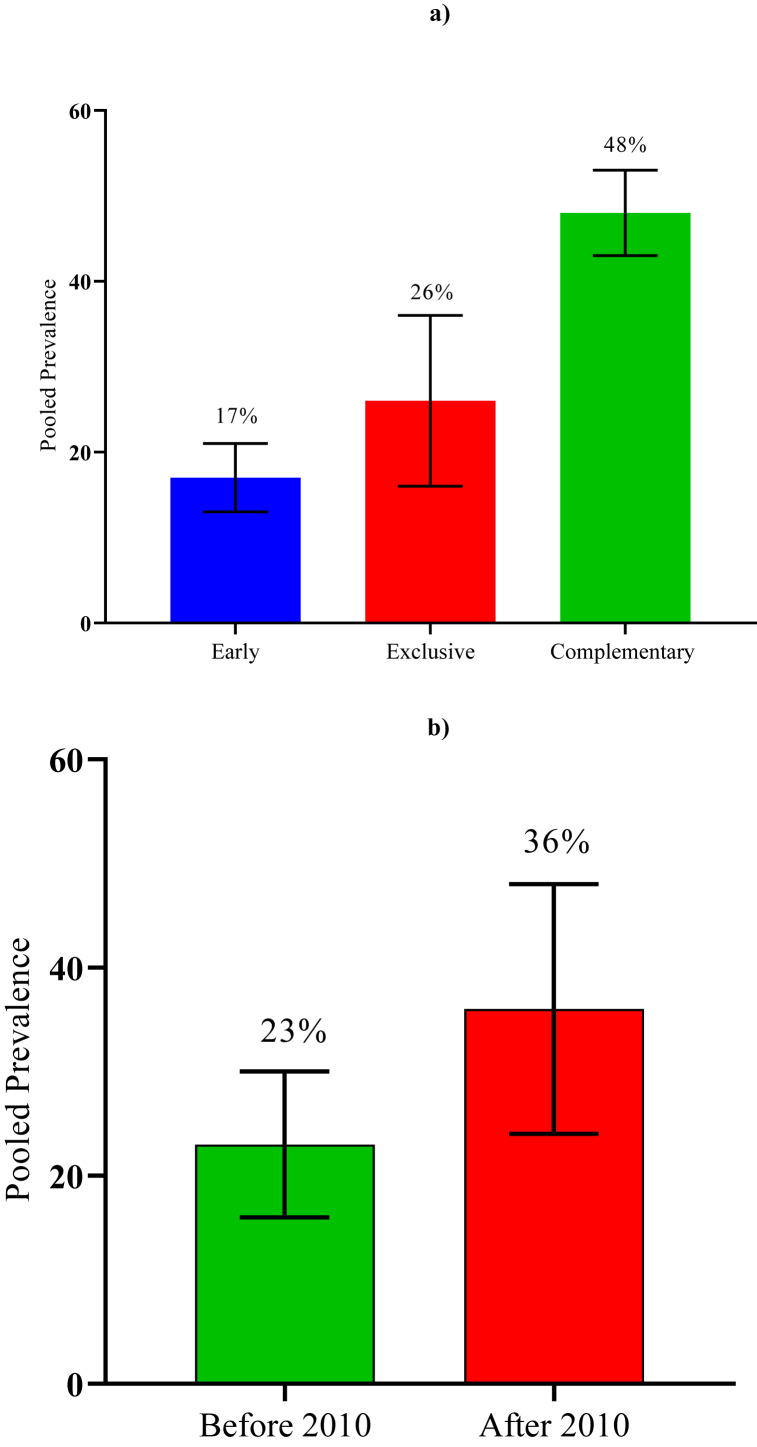
a) Prevalence of PND among Bangladeshi women in various phases*; b)* Trends of postnatal depression.

“PND” among Bangladeshi mothers of different ages of their infants was determined through the differentiation of studies with different targeted populations. The studies on “PND” were divided into three stages named depression of mothers at early, exclusive, and complementary age of the child. The early-stage pooled prevalence was 17.12 % (95 % CI: 13.14 % ⎯ 21.1 %, I^2^: 86.46 %) [16,33,[41], [42], [43],^45^]. The clustered prevalence among mothers at the exclusive age of the child was 25.73 % (95 % CI: 18.74 % ⎯ 32.73 %, I^2^:97.67 %) [34,36,39,40,43,45,47,49], whereas the shared prevalence among mothers at the complementary age of the child was 48.11 % (95 % CI: 42.85 % ⎯ 53.4 %, I^2^:60.95 %) [35,39,46,48]. The results indicate that the prevalence rate was found to be higher with the time of the postnatal period (Fig. 4a).

### 3.5 Trends of “PND” across the decades of the 21st century

The postnatal depression (PND) trend among mothers in Bangladesh was determined by using estimated prevalence from studies that collected their data before and after 2010 (Fig. 4b). In this sub-group analysis, 10 studies [16,36,[39], [40], [41], [42], [43], [44], [45],^49^] were included that collected their data before 2010. Six studies [[33], [34], [35],[46], [47], [48]] collected their data after 2010. The pooled prevalence of studies on PND in the 2000s was 22.78 % (95 % CI: 17.82 % ⎯ 27.73 %, I^2^ > 96.65 %). The result was dramatically changed after 2010. The pooled prevalence of studies on PND in the 2010s was 36.00 % (95 % CI: 23.94 % ⎯ 48.06 %, I^2^ > 95.76 %).

### 3.6 Publication bias

**Fig. 5:**
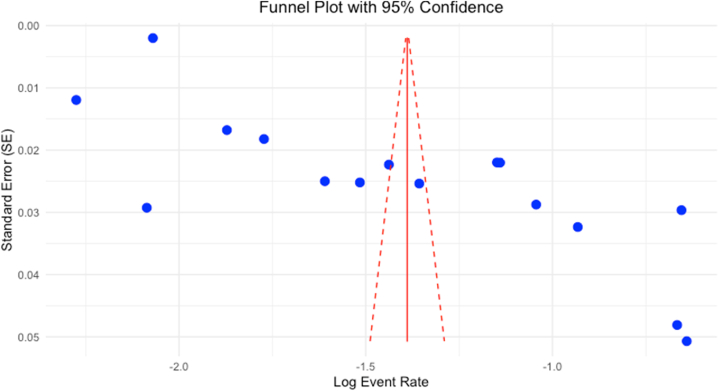
Funnel plot showing asymmetrical distribution of included studies.

There is a possibility of publication bias in meta-analysis. To investigate publication bias, funnel plots were applied to visually inspect the asymmetry in the distribution of study effect sizes against their standard errors. An asymmetrical inverted funnel plot indicates a high likelihood of publication bias (Fig. 5). Alongside the visual assessment, Egger's and Begg's tests were employed to provide supplementary evidence. A p-value of less than 0.005 (p < 0.0001) suggests significant publication bias in the included studies (B = −2.0232, SE = 0.0546, p < 0.0001), supporting the visual assessment.

### 3.7 Quality assessment of included study

In quality-based sub-group analysis of “PND”, a total of six studies were found to a high-quality studies, and the estimated pooled prevalence was 19.55 % (95 % CI: 13.94 % ⎯ 25.16 %, I^2^ > 96.91 %) [36,40,41,44,45,49]. On the other hand, 10 studies were defined as low quality according to our assessment criteria, and the observed pooled prevalence was 33.04 % (95 % CI: 24.96 % ⎯ 41.12 %, I^2^ > 94.95 %) [16,[33], [34], [35],^39^,^42^,^43^,[46], [47], [48]].

### 3.8 Associated factors of “AND” and “PND”

Several studies indicated that intimate partner violence, including physical violence, emotional violence, and sexual violence, is significantly associated with postnatal depression [35,40,41,46,47,49]. Along with the violence to women, unsupportive spouse, mother-in-law, and lack of familial and social support also increased both antenatal and postnatal depression among mothers [16,33,37,49]. Additionally, two studies reported that unplanned pregnancy was one of the risk factors for depression among mothers [35,36] Cited in Table 3.

**Table 3:** Antenatal depression (AND) and Postnatal depression (PND) prevalence in Bangladesh: A summary of associated factors with Antenatal depression (AND) and Postnatal depression (PND).

Author; Year	Associated risk factors
Nasreen et al., 2010 [38]	Older age, less education, lower body weight, low BMI, low SES, low maternal nutrition.
Gausia et al., 2009 [37]	History of beaten by husband, unhelpful or unsupportive mother-in-law, husband, or family preference of the male child.
Edhborg et al., 2011 [41]	Older, less educated, intimate partner violence, lower emotional bonding with their infants due to girl.
Azad et al., 2019 [35]	Uneducated women, Job involvement after child delivery, left the job after pregnancy, miscarriage or stillbirth or child death, unplanned pregnancy, the family, arranged the delivery cost by borrowing, selling or through mortgage assets, perceived antenatal stress, at least one EPDS depressive symptom developed during the pregnancy period, higher for women who sometimes/never/rarely shared their personal feelings with their husband, intimate partner violence
Sharmin et al., 2019 [34]	Malnutrition, lower growth, depressed mothers were ill and seeks treatment from the hospital.
Parveen et al., 2017 [33]	Maternal age, history of depression, lack of social support
Edhborg et al., 2020 [40]	Physical violence, Emotional violence, Sexual violence, IPV, Mother's perception of infant's temperament
Surkan et al., 2018 [36]	Unwanted pregnancy (postnatal), women who perceived their husbands didn't want pregnancy; both parents who did not want pregnancy had depressive syndrome for the prenatal and postnatal stage. Maternal age more than 30, poor SES, low maternal education
Gausia et al., 2009 [16]	Post mental illness, depression in the current pregnancy, perinatal death, and poor relationship with the mother-in-law, either the husband or the wife leaving home after a domestic quarrel
Black et al., 2009 [39]	Maternal education, poverty, infant temperament
Kabir et al., 2014 [49]	Poor relationship with their husbands and mother-in-law, physical violence from their spouses during their marriage, physical violence by their partner during pregnancy, forced to have sex against their will 6–8 months after childbirth, emotional violence by their intimate partner
Hamadani et al., 2012 [45]	Child mental development
Gausia et al., 2012 [44]	Negative experience of childbirth significantly associated with PND.
Islam et al., 2017 [47]	Physical, sexual, and psychological IPV both during pregnancy and after childbirth were significantly associated with PPD
Jiang et al., 2017 [48]	Maternal depression was associated with lower cognitive development or lower neurodevelopment.
Gausia et al., 2011 [43]	Prenatal death was significantly associated with PND
Gausia et al., 2010 [42]	Diarrhea, malnutrition among infants.
Hossain et al., 2020 [46]	Maternal age, no family food security, violence against woman

## 4 Discussion

### 4.1 Prevalence of “AND” and “PND”

This review measured the prevalence of “AND” and “PND” and their associated factors in Bangladesh. This meta-analysis found that the pooled prevalence of “AND” and “PND” among Bangladeshi women were 19.5 % and 27.75 %, respectively. Similar studies on depression in Turkey and Pakistan found a close pooled prevalence of “PND” of 24 % and 30 %, respectively [50,51]. The “PND” prevalence similarity with Asian countries like Pakistan and Turkey might have been due to their homogeneous demographic and socioeconomic characteristics. Additionally, a study in Ethiopia stated a “PND” prevalence of 22.08 %, which aligns with the study finding [52]. An Iranian and Indian meta-analysis found that the pooled prevalence of “PND” was 25.3 % and 22 %, which is close to our study finding [53,54]. However, high-income countries like Denmark had a significantly lower “PND” prevalence (15 %) than the current study finding [55]. The discrepancy between low-middle-income countries and high-income countries in the prevalence of “PND” might be due to a lack of awareness of “PND” among healthcare professionals in low-middle-income countries that leads to late recognition and management of “PND” [56,57].

Moreover, due to cultural and religious variations, other Southeast Asian countries, Singapore (9.8 %) [58], Timor Leste (12.6 %) [59], and Vietnam (8.5 %) [60] had comparatively lower prevalence of “PND” than Bangladesh, Pakistan, and India. A recent systematic qualitative study identified factors like limited resources allocated for mental health, stigma, lack of awareness, and shame preventing adequate care on perinatal health for women with perinatal mental health conditions in these regions [61]. Targeted interventions include increasing awareness among women to destigmatize maternal depression and training and educating healthcare providers to manage maternal depression during antenatal and postnatal visits of mothers.

A scoping review found that the prevalence of AND ranges from 4.9 % to 46.8 % among the mothers of Southeast Asian countries [11]. The study was conducted in Pakistan [51]. However, the current study found a relatively lower prevalence of “AND” compared to Pakistan, which might result from the low numbers of studies conducted in Bangladesh on “AND."

### 4.2 PND prevalence increases as children grow

This study measured that the prevalence of “PND” increases as the child grows up among mothers. A study found that mothers of formula-feeding children have experienced more depression than mothers of exclusive-breastfeeding children [62]. Mothers might maintain breastfeeding at the early and exclusive breastfeeding stages, leading to low EPDS scores rather than a complementary stage in our study, like other studies [63,64]. Another explanation could be that mothers face depression-influencing factors like the need for complementary food for their child after the exclusive breastfeeding stage that may influence “PND” symptoms. Several studies have shown no significant association between breastfeeding status and “PND” [[65], [66], [67]]. It is still unclear whether there is an association or not between breastfeeding and depression. Future studies should focus on discovering the association between breastfeeding and “PND".

### 4.3 Secular trend

One of the key findings of the current study was to compare the “PND” prevalence of the 2000s and 2010s. “PND” prevalence among women in the 2010s is approximately 13 % higher than in previous decades. A consistent development in the socioeconomic sector has been observed in Bangladesh in the last decade, which has significantly changed cultural attitudes, economic conditions, and social factors like health-seeking behavior, healthcare access, mental health awareness, and better reporting [68,69]. These could be the potential reasons for the increasing prevalence of “PND” over time in Bangladesh.

Moreover, a recent study revealed high frequency of antenatal care visits, high family income, income dissatisfaction, C-section delivery, pregnancy complications, and high childbirth expenses were associated with an increase in “PND” and recommended targeted interventions to support these vulnerable groups of people [70]. Similar studies should explore broader social determinants (healthcare access, mental health awareness, economic pressures, and societal support systems) of “PND”. By considering these aspects, researchers may better understand contextual factors driving the increasing prevalence of “PND”. Early intervention strategies like increasing awareness of antenatal visits, improved screening of depression at the perinatal period of women, and support system for postpartum women could be the key strategies to reduce the burden of “PND” in this region. A similar finding was shown by a study in England [71] that compared depression between two generations. Future studies could measure depression status among women generation-wise.

### 4.4 Associated factors of “PND”

We have identified several factors associated with “PND,” as represented in Table 3. Some of the significant associated factors were unplanned pregnancy, different intimate partner violence (physical violence, emotional violence, and sexual violence), male child preference, and bitter relations with husband and mother-in-law. Several studies are compatible with our findings. Unwanted pregnancy [36,52], history of violence [72,73], unsupportive husband [74], and male gender preference [75] were significantly associated with the increased likelihood of depression among mothers. Family support to the mothers who can talk and interact with others and are satisfied with the pregnancy was significantly associated with the decreased likelihood of depression [52,75].

National Mental Health Act 2018 imparts the integration of mental health into the primary healthcare system. It ensures protections and rights for people suffering from mental health along with access to mental healthcare. Moreover, promoting early detection of maternal depression, reducing stigma, and increasing awareness got priority in the National Mental Health Strategic Plan 2020–2030. The effective implementations of these policies at the community level are crucial to reducing the existing burden of maternal depression during and after pregnancy [27,28]. The rising trend we found in maternal depression could inform the effective implementation of the above-prioritized policies. For instance, these findings could be used to demonstrate the importance of screening during antenatal and postnatal visits allowing for early detection. Additionally, we can highlight these findings in public health campaigns to increase awareness, and reduce social stigma associated with maternal depression.

Most published studies included in this review were either cross-sectional or cohort designs. There is a lack of community-based interventional studies that would be particularly valuable in exploring which interventions are effective in reducing the burden of maternal depression. Future studies can provide more robust evidence to reform public health policy and improve health outcomes.

### 4.5 Strengths and limitations

This is the first systematic review and meta-analysis conducted to summarize the prevalence of “AND” and “PND” among Bangladeshi women. These findings provide essential baseline information that can inform future research and serve as a valuable reference point for predicting the prevalence of perinatal depression in similar lower-middle-income countries. The prevalence (27.75 %) is comparatively higher than the global prevalence of “PND”, which was 17.2%–17.7 % [12,13]. This indicates an alarming statement and the need for emergency actions by government and health policymakers to control and reduce the burden of “PND".

This study used PRISMA guidelines [76] that ensure a systematic and thorough approach to identifying relevant studies. We used different national and international databases, and two independent investigators evaluated the quality of the articles to reduce investigator bias and show maternal “PND” prevalence varied by the study quality. This study estimated pooled prevalence along with the associated factors of postnatal depression and antenatal depression.

Study population, study designs (cross-sectional study design and cohort study design), measurement techniques of depression (EPDS, CESD, Self-reporting questionnaire and PHQ), and follow-up periods across studies could contribute to the heterogeneity. Thus, data interpretation should be done cautiously as very high heterogeneity (between study variability) and publication bias were observed. A random effect model was used to adjust the variability. In addition, subgroup analysis (quality of the study, decade-based analysis, and PND at different stages of a child) was also used to explore the sources of heterogeneity. However, in the subgroup analysis, a disproportional number of studies could minimize the precision of the estimated value.

Moreover, few studies were included for AND, which might overestimate or underestimate the pooled estimated prevalence of AND in Bangladesh. There were some other limitations, such as sensitivity analysis not being performed for this systematic review, which may impact the overall quality of the review. These limitations should be considered when interpreting the findings. Only articles published in English were included in this study, which might have resulted in excluding studies published in other languages. This could affect the comprehensiveness and generalizability of the findings.

## 5 Conclusion

The study found that the pooled prevalence of “AND” and “PND” is 19.5 % and 27.75 %, respectively. The increasing prevalence of “AND” and “PND” was observed, and the trend was significantly higher during the 2010s (36.0 %) than in the previous decade (22.78 %) in Bangladesh. The depression among Bangladeshi mothers during pregnancy and after childbirth needs more attention. Screening of depression during the perinatal period needs to be included as one of the family planning and mental health services facilities. The identified associated factors could be targeted for evaluation, prevention, interventions, and management of “AND” and “PND” among Bangladeshi mothers.

## CRediT authorship contribution statement

**Mohammad Injamul Hoq:** Writing – review & editing, Supervision, Methodology, Formal analysis, Data curation. **Md Mohotasin Hossain:** Writing – original draft, Methodology, Formal analysis, Data curation, Conceptualization. **Mohammad Aktar Sayeed:** Writing – review & editing, Supervision. **Md Jakaria:** Writing – review & editing, Writing – original draft, Supervision.

## Data availability statement

Data included in the article/supplementary material is referenced in the article.

## Ethical consideration

None.

## Funding

This research received no specific grant from funding agencies in the public, commercial, or not-for-profit sectors.

## Declaration of competing interest

The authors declare the following financial interests/personal relationships which may be considered as potential competing interests:Mohammad Injamul Hoq reports statistical analysis was provided by Drug Insides & Disease Epidemiology, Chattogram, Bangladesh. If there are other authors, they declare that they have no known competing financial interests or personal relationships that could have appeared to influence the work reported in this paper.
