# Supplementary material for: Trends in the prevalence of antenatal and postnatal depression in Bangladesh: A systematic review and meta-analysis
Source: Heliyon. 2025 Jan 14;11(2):e41955. doi: 10.1016/j.heliyon.2025.e41955 (PMC11787471; doi:10.1016/j.heliyon.2025.e41955)
Supplement: Multimedia component 4 [file mmc4.pdf]

BMC Research Notes

TECHNICAL NOTE Open Access

Meta-analyses and Forest plots using a microsoft excel spreadsheet: step-by-step guide focusing on descriptive data analysis

Jeruza L Neyeloff[1*], Sandra C Fuchs[1,2] and Leila B Moreira[1,2]

Abstract

Background: Meta-analyses are necessary to synthesize data obtained from primary research, and in many situations reviews of observational studies are the only available alternative. General purpose statistical packages can meta-analyze data, but usually require external macros or coding. Commercial specialist software is available, but may be expensive and focused in a particular type of primary data. Most available softwares have limitations in dealing with descriptive data, and the graphical display of summary statistics such as incidence and prevalence is unsatisfactory. Analyses can be conducted using Microsoft Excel, but there was no previous guide available.

Findings: We constructed a step-by-step guide to perform a meta-analysis in a Microsoft Excel spreadsheet, using either fixed-effect or random-effects models. We have also developed a second spreadsheet capable of producing customized forest plots.

Conclusions: It is possible to conduct a meta-analysis using only Microsoft Excel. More important, to our knowledge this is the first description of a method for producing a statistically adequate but graphically appealing forest plot summarizing descriptive data, using widely available software.

Background

Meta-analyses and systematic reviews are necessary to synthesize the ever-growing data obtained from primary research. Performing a search on Pubmed limiting to the type of article, the Mesh term “meta-analysis” will wield 4223 results in 2010 only. Although reviews of interventional studies, especially clinical trials, provide the best evidence, there are several situations in which observational studies are the only alternative. Meta-analyses of these studies are becoming more common, particularly after publication of the MOOSE statement [1]. Some of the studies are not concerned with the assessment of relative risks or odds ratios, but are focused on a summary statistics of incidence or prevalence.

General purpose statistical packages such as SPSS, Stata, SAS, and R can be used to perform meta-analyses, but it is not their primary function and hence they all require external macros or coding. These can be downloaded, but are not always easy for the researcher to understand or customize. Additionally, the first three programs do not have free access, with prices ranging from $250 to over $30,000 depending on version and country. R is a very resourceful open source package, but its use in health is still limited, due mostly to the need of programming instead of a point-and-click interface.

There are some software packages specifically developed to conduct meta-analyses. RevMan [2] is a freeware program from the Cochrane Collaboration that requires the researcher to fill all steps of a systematic review. It only accepts effect sizes in traditional formats. Metawin [3] and Comprehensive Metanalysis (CMA) [4] are commercial software that have user friendly interfaces. The former only accepts three types of primary data, while the latter has a purchase cost, but accepts more types of data. It can perform advanced analyses, but there are still limitations regarding graphic display, particularly of descriptive data, since CMA does not allow customization of the forest plot produced. Finally, there is also Meta-Analysis Made Easy (MIX) [5], an

* Correspondence: jeruza_med@yahoo.com.br
[1]Post Graduate Program of Cardiology, Universidade Federal do Rio Grande do Sul, Porto Alegre, Brazil
Full list of author information is available at the end of the article

© 2012 Neyeloff et al; licensee BioMed Central Ltd. This is an Open Access article distributed under the terms of the Creative Commons Attribution License (http://creativecommons.org/licenses/by/2.0), which permits unrestricted use, distribution, and reproduction in any medium, provided the original work is properly cited.

add-on for Excel. It can be used for analysis of descriptive data selecting the input type to "continuous", but the free version does not allow for analysis of original data, only build in datasets. Some other options are no longer available, as FAST*PRO [6], and others are still currently under development, as Meta-Analyst [7].

Another option would be to analyze data using directly Microsoft Excel. Although it has a purchase cost, it is usually already installed in most computers, bundled with Microsoft Office package. Most researchers would be uncomfortable entering all the formulas themselves, since they may seem complex at first. However, if the calculations are done in steps, statistics like Q and I^2 can be computed with basic arithmetic operations. Borestein et al [8] cites the impossibility of producing forest plots as an important limitation, but we have developed a method to turn a scatter plot into a statistically correct forest plot, allowing the researcher to take advantage of all excel formatting tools. Our work is separated into two spreadsheets, so researchers can use both to conduct all calculations or simply the second one if they have already analyzed the data in any other software, but want an appealing graphical way of presenting it [Additional file 1].

Findings

Technical notes

The method described here was designed on a laptop with Intel Core Duo 2.2 GHz processor, 4 GB RAM, running Windows Seven 64 bit and Microsoft Office Excel 2007. The spreadsheets were later tested on Excel 2003, with no differences found in either the calculations or graphs.

The outcome of meta-analyses is the effect summary. However, some reviews may only aim in combining rates or prevalences; technically these cannot be called "effects", since there is nothing "causing" it, and the correct term would be single group summary. We will refer to both these estimates simply as "outcome" in order to avoid confusion, and maintain only the abbreviation as *es* to follow textbooks standard.

Since we have established that the limitation of the existing software packages is handling descriptive data, we will be using rates in our example so that the difference in the final forest plot is more overt. The data could be the prevalence of smoking in a country or the incidence of myocardial infarction in high risk patients. We chose to use theoretical numbers so we could openly distribute the spreadsheets, test particular formulas and compare results obtained with other software. All formulas are presented in traditional equations and also in excel format.

Steps 1 and 2 always require adjustments according to study type and outcome. Columns in light grey in spreadsheet 1 are the ones to be adapted, while columns in dark grey do not require any modification regardless of study type (this includes all further steps of the guide). The necessary adjustments can be easily found on methodological books [8-10].

Cell B14 should be filled with the number of studies being analyzed. There are annotations on the spreadsheet that pop up when the mouse pointer is upon selected cells, so the downloaded file can be used without constant consultation of the full article. The explanation for the formulas and detailing of steps are not present on the spreadsheet though. A recently published paper by Schriger et al [11] reviewed over 300 systematic reviews and highlighted important aspects of producing forest plots, which were considered in developing this approach.

Steps in analyzing data and producing a forest plot

Spreadsheet 1-analysis (Figure 1)

1. Calculating the outcome (effect size, es)

In our example we have the number of events and the number of subjects in columns B and C, so we can simply compute the rate in column D as $\frac{n_{events}}{n_{total}}$ or *D*3 = *B*3/*C*3 in Excel. It is the same from D3 to D12, and copy and paste will automatically adjust the cell numbers. This copying and pasting should be done for steps 1 through 6 and in step 9 B.1.

2. Calculating Standard Error (SE)

All SE can be derived from the formula $\mathrm{SE} = \frac{\sum(\bar{x}-\mu)^2}{\sqrt{n}}$, but there are simplified derived equations for different types of studies. Since we are using rates, we can use $\mathrm{SE} = \frac{es}{\sqrt{es*n}}$ or $\mathrm{SE} = \frac{\sqrt{events}}{n}$, the same formula used in CMA. In excel this will be *E*3 = *D*3/SQRT(*D*3*C3).

3. Computing variance (Var)

This formula is simple: Var = SE^2. In Excel, *F*3 = *E*3^2.

4. Computing individual study weights (w)

We must weight each study with the inverse of its variance, so $w = \frac{1}{\mathrm{SE}^2}$ or *G*3 = 1/*F*3 in Excel.

5. Computing each *weighted effect size* (w*es)

This is computed multiplying each effect size by the study weight. If we are not using any corrections on the weight (meaning, single effect model) this equation will result again in the study size for some types of studies. In excel, this will be *H*3 = *G*3**D*3

6. Other necessary variables (w*es^2 and w^2)

We will need two other variables in order to calculate the Q statistics (columns I and J of spreadsheet 1). In excel this will be *I*3 = *G*3*(*D*3 ^ 2) and *J*3 = *G*3 ^ 2.

	A	B	C	D	E	F	G	H	I	J	K	L	M	N	O
1	Theoretical Study: Prevalence of variable A in population B - metanalysis of 10 cross sectional studies.														
2	Study	Events	Sample Size	Outcome	SE	Var	w	w*es	w*(es^2)	w^2		w_v	w_v*es	w_v*(es^2)	w_v^2
3	Author 1, year	404	2002	0.2017982	0.010039836	0.000100798	9920.802	2002	404	98422311.93		5028.223353	1014.6864	204.761897	25283030
4	Author 2, year	58	353	0.1643059	0.021574428	0.000465456	2148.431	353	58	4615755.91		1774.512529	291.56297	47.9055297	3148894.7
5	Author 3, year	187	944	0.1980932	0.014486011	0.000209845	4765.433	944	187	22709353.16		3247.558672	643.31936	127.437203	10546637
6	Author 4, year	258	1410	0.1829787	0.011391758	0.000129772	7705.814	1410	258	59379568.69		4388.828349	803.06221	146.943298	19261814
7	Author 5, year	451	2103	0.2144555	0.010098317	0.000101976	9806.228	2103	451	96162115.07		4998.622741	1071.9823	229.892551	24986229
8	Author 6, year	430	2420	0.177686	0.008568777	7.34239E-05	13619.53	2420	430	185491730.4		5830.800215	1036.0513	184.091756	33998231
9	Author 7, yeat	669	3100	0.2158065	0.008343559	6.9615E-05	14364.72	3100	669	206345280.3		5963.239572	1286.9056	277.722525	35560226
10	Author 8, year	52	251	0.2071713	0.028729492	0.000825384	1211.558	251	52	1467872.042		1082.880625	224.3418	46.4771862	1172630.4
11	Author 9, year	1535	7790	0.1970475	0.005029406	2.52949E-05	39533.62	7790	1535	1562906765		8105.433602	1597.1554	314.715474	65698054
12	Author 10, year	241	1274	0.189168	0.01218538	0.000148483	6734.755	1274	241	45356927.43		4055.765202	767.22089	145.133622	16449231
13															
14	k	10				Sums:	109810.9	21647	4285	2282857680		44475.86486	8736.2882	1725.08104	236104979
15	df	9													
16												v	9.808E-05		
17	Q	17.731188			Q_v	9.032742172									
18	I^2	49.241981			I^2_v	0.362483188									
19															
20	es (fixed)	0.1971298			es (random)	0.196427619									
21	SEes (fixed)	0.0030177			SEes (random)	0.004741741									
22	CI (fixed)	0.1912151	0.20304451		CI (random)	0.187133807	0.205721								

Figure 1 Spreadsheet 1: Analysis This spreadsheet contains the calculations necessary for the analyses. Input in light gray columns must be adapted according to effect size type. Calculations in dark grey columns are the same for any effect size type.

Now we need to sum all values of each variable. In our spreadsheet they are in line 14, labeled "Sums": *G*14 = *SUM* (*G*3:*G*12), *H*14 = *SUM* (*H*3:*H*12), *I*14 = *SUM* (*I*3:*I*12), *J*14 = *SUM* (*J*3:*J*12)

7. Calculating Q

The Q test measures heterogeneity among studies, and works like a t test. It is calculated as the weighted sum of squared differences between individual study effects and the pooled effect across studies, with the weights being those used in the pooling method. Q is distributed as a chi-square statistic with k (number of studies) minus 1 degrees of freedom. Our null hypothesis is that all studies are equal. To test that, we need to calculate Q and compare it against a table of critical values. If our calculated Q is lower than that of the table's, than we fail to reject the null hypothesis (and hence the studies are similar).

The formula is $Q = \sum (w^*ES^2) - \frac{[\sum (w^*ES)]^2}{\sum w}$, but in our spreadsheet it will be simply *B*17 = *I*14 - ((*H*14 ^ 2)/*G*14) since we already have all the sums.

8. Calculating I^2

The I^2 was proposed as a method to quantify heterogeneity, and it is expressed in percentage of the total variability in a set of effect sizes due to true heterogeneity, that is, to between-studies variability. The formula is $I^2 = \frac{(Q - df)}{Q} * 100$, where "df" stands for "degrees of freedom", simply the total number of studies (k) minus 1. In excel, *B*18 = ((*B*17 - *B*15)/*B*17)*100.

9. Deciding on effect summary $(\overline{es})$ model.

If heterogeneity is low, we can use a fixed effect model, that assumes the effect size is the same in our parameter population, and differences in studies are just from sampling error. However, if we think our sample populations may differ from each other, we can use a random effects model. Many researchers will choose this model even if heterogeneity is low. In our example, Q is higher than 16.919, the critical value for 9 degrees of freedom found in a chi-square distribution, and I^2 is 49%, so we have moderate heterogeneity [12]. We must decide whether the data is possible to meta-analyze, and if so we may choose to proceed to a random effects models.

A. Fixed effects Model

Our effect summary is $\overline{es} = \frac{\sum (w^*es)}{\sum w}$, or *B*20 = (*H*14/*G*14). The standard error is $SE_{\overline{es}} = \sqrt{\frac{1}{\sum w}}$, or *B*21 = *RAIZ* (1/*G*14). With the $SE_{\overline{es}}$ we calculate the 95% Confidence Interval, as $CI\,(\overline{es}) = \overline{es} \mp 1,96 * SE$. In Excel, *B*22 = *B*20 - (1.96**B*21) and *C*22 = *B*20 - (1.96**B*21). In our example we will not use these results.

B. Random effects model

Since we are assuming that variability is not only due to sampling error, but also to variability in the population of effects, in this model the weight of each study will be adjusted with a constant (*v*) that represents this.

B1. The formula is $v = \frac{Q - (k-1)}{\sum w - \left(\frac{\sum w^2}{\sum w}\right)}$. We have all these information, except for $\sum w^2$. We can compute w^2 in column J with *J*3 = *G*3 ^ 2, and then its sum with J14 = *SOMA* (*J*3: *J*12). Now, applying the formula, *M*16 = (*B*17 - *B*15)/(*G*14 - (*J*14/*G*14)).

	A	B	C	D	E	F	G	H	I	J	K
1	Study	Events	Sample Size	Outcome	SE	CI lower	CI upper		Rate	CI lower	CI upper
2	Author 1, year	404	2002	0.2017982	0.010039836	0.1821201	0.2214763	11	20.17982	1.9678078	1.9678078
3	Author 2, year	58	353	0.1643059	0.021574428	0.1220201	0.2065918	10	16.430595	4.2285879	4.2285879
4	Author 3, year	187	944	0.1980932	0.014486011	0.1697006	0.2264858	9	19.809322	2.8392581	2.8392581
5	Author 4, year	258	1410	0.1829787	0.011391758	0.1606509	0.2053066	8	18.297872	2.2327845	2.2327845
6	Author 5, year	451	2103	0.2144555	0.010098317	0.1946628	0.2342482	7	21.445554	1.9792701	1.9792701
7	Author 6, year	430	2420	0.177686	0.008568777	0.1608911	0.1944808	6	17.768595	1.6794804	1.6794804
8	Author 7, yeat	669	3100	0.2158065	0.008343559	0.1994531	0.2321598	5	21.580645	1.6353377	1.6353377
9	Author 8, year	52	251	0.2071713	0.028729492	0.1508615	0.2634811	4	20.717131	5.6309805	5.6309805
10	Author 9, year	1535	7790	0.1970475	0.005029406	0.1871899	0.2069051	3	19.70475	0.9857637	0.9857637
11	Author 10, year	241	1274	0.189168	0.01218538	0.1652846	0.2130513	2	18.916797	2.3883346	2.3883346
12	Summary			0.196374	0.0047	0.187162	0.205586	1	19.6374	0.9212	0.9212

	B	C
14	Central Tendency	
15	0	19.6374
16	1	19.6374
17	2	19.6374
18	3	19.6374
19	4	19.6374
20	5	19.6374
21	6	19.6374
22	7	19.6374
23	8	19.6374
24	9	19.6374
25	10	19.6374
26	11	19.6374
27	12	19.6374

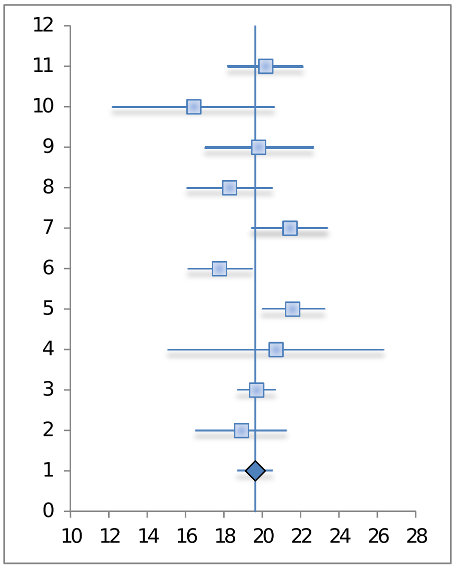

Figure 2 Spreadsheet 2: Forest Plot This spreadsheet contains the final forest plot. Data must be manually entered, either after using spreadsheet 1 or any other analysis software.

B2. Once we have the constant, we can calculate new weight for each study, using $w_v = \frac{1}{(SE^2 + v)}$. In excel, *L*3 = 1/((*E*3 ^ 2)+$*M* $16). We need the $ to fix cell M16, or else it will change when we copy the equation to cells L4 to L12.

B3. Now we repeat steps 5 to 8, but using our new weight W_v. The results are in columns M, N and O. Applying the Q and I^2 formulas we have now an acceptable Q and low heterogeneity. We calculate our effect summary as $\overline{es}_v = \frac{\sum (w_v * ES)}{\sum w_v}$, and standard error as $SE_{\overline{es}_v} = \sqrt{\frac{1}{\sum w_v}}$.

In excel: *F*20 = *M*14/*L*14, *F*21 = *SQRT* (1/*L*14), *F*22 = *F*20 - (1.96**F*21) and *G*22 = *F*20+(1.96**F*21). The confidence intervals are broader than the ones calculated with fixed effect model, however, little change in the effect summary is expected.

Analyzing these numbers in CMA we achieved exactly the same results. - [Additional files 2 and 3].

Spreadsheet 2-forest plot (Figure 2)

Columns A-G have the studies information. The user can insert each study effect size and confidence interval directly into columns D, F and G if he has the data. In our example we copied the calculations from spreadsheet 1, and also the values of the random effects model effect summary.

1. Make sure the information is the way we want it displayed. In our example, we wanted the rates in percentages, so column I = column D*100.

2. We usually read the lower and upper confidence interval as a value, but excel understands it as a difference to the mean. This is key to obtain a proper forest plot. These values are *J*2 = *I*2 - (100**F*2) and *K*2 = *I*2 +

(100* *F*2). Again, we multiply by 100 to have it in percentage.

3. In order to have each study in a different line, we will assign ordinal numbers to the studies. Our effect summary must be number 1 if we want it in the bottom of the graph. This is done manually in column H of our spreadsheet.

4. We are ready to build the graph. Insert > Graph > Scatter Plot. X values will be column I, lines 2-12, and Y values column H, lines 2-12.

5. We must now add the error bars. In Excel 2007 this is done in the Layout tab, clicking the "Error Bar" button on the right side. In Excel 2003 we must right click on the data series (points on the graph) and click "format data series", then chose the "X error bar" tab. In this window we mark the option "personalized values", and then assign columns J and K, lines 2 to 12, to the lower and upper value.

6. To insert the line marking the summary effect value we will add another data series. First we manually build this data set in the spreadsheet. Then right click on the graph > Select Data. Click on "add", and chose X values as column C, lines 15 to 26, and Y values as columns B, lines 15 to 26. A new set of points will appear on the graph. Right-click on any of the new dots and select "format data series". Then we will choose "no marker" and "solid line" on the Marker Options and Line Color tabs.

7. We can now format the X axis, right-clicking on it. In our example we want it to begin on 10 and end on 28, interval of 2 units. It is not our case, but if the researcher is dealing with relative data, then "logarithmic scale" must be marked.

8. The graph is ready. The user can format colors, outlines, shadows and sizes. In our example we changed the summary effect to a diamond shape. This is done by selecting only one dot (double click) and then right clicking it.

9. For presentation we recommend copying and pasting the graph over a table with study information (Figure 3).

Conclusion

We have constructed a guide to aid researchers interested in meta-analyzing data using a spreadsheet. To the best of our knowledge there is no prior step-by-step approach, but it should be noted that all formulas and methodology were previously publicly available.

The main limitation of analyzing data in a spreadsheet is the potential for errors by typing incorrect formulas. We believe that a step-by-step approach as those presented in this article with all formulas already incorporated in the excel format can help minimize this possibility. The guide presented also does not handle advanced analyses such as multiple regression. However, this is not frequently used in summarizing descriptive data. All sensitivity analysis must be done manually, including and excluding each study of the effect summary calculations, but this limitation is also present in other softwares.

Microsoft Excel is part of the Microsoft Office Package, and therefore it is not free of costs. However, for those who already have the package, this use of Excel could amplify its utility offering an alternative for customizing the graphic presentation of the forest plot.

The main limitation of the forest plot is that all studies are represented by squares of the same size, instead of proportional to study weight. We did not feel this could overshadow all other formatting possibilities, since study weight can also be estimated by the confidence interval width.

In conclusion, it is possible to meta-analyze data using a Microsoft Excel spreadsheet, using either fixed effect or random effects model. The main advantages of this approach are the understanding of the complete process

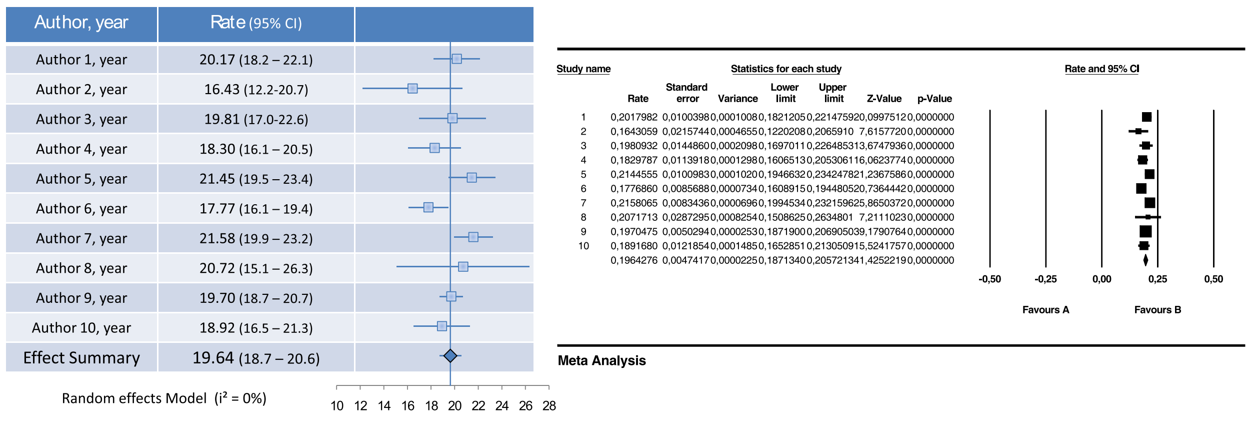

Figure 3 Comparison of Forest Plots Comparison of forest plots produced using our spreadsheet (left) and CMA (right).

and formulas, and the use of widely available software. It is also possible and simple to make a forest plot using excel. Since displaying results in a graphically appealing but also statistically correct way is usually a problem to most researchers, we believe the method presented here could be of great use. Figure 3 compares the graph obtained with our method and with CMA software.

Availability and requirements

Project name: Meta-analyses and Forest Plots using a Microsoft Excel spreadsheet: step-by-step guide focusing on descriptive data analysis;

Project home page: none;

Operating systems: any OS supporting Microsoft Excel;

Programming language: not-applicable;

Other requirements: Microsoft Excel 2003 or higher;

License: Creative Commons Attribution 3.0 Unported (CC BY 3.0);

Restrictions to use by non-academics: none

Availability of supporting data

The spreadsheets mentioned and the CMA files used for comparison of statistics are available as complementary material.

Additional material

Additional file 1: Meta-analyses and forest plots in MS Excel. This file contains both spreadsheets developed.

Additional file 2: CMA calculations fixed effect. This is a portable document format (pdf) of the calculations performed by the software Comprehensive Meta-Analysis, when calculating the effect summary using fixed effect model. It is provided so readers may compare the calculations and results obtained using Microsoft Excel spreadsheet and the commercial software.

Additional file 3: CMA calculations random effects. This is a portable document format (pdf) of the calculations performed by the software Comprehensive Meta-Analysis, when calculating the effect summary using random effects model. It is provided so readers may compare the calculations and results obtained using Microsoft Excel spreadsheet and the commercial software.

Acknowledgements

This study was funded by Conselho Nacional de Pesquisas (CNPq) and Fundo de Incentivo à Pesquisa do Hospital de Clínicas de Porto Alegre (FIPE-HCPA).

Author details

[1]Post Graduate Program of Cardiology, Universidade Federal do Rio Grande do Sul, Porto Alegre, Brazil. [2]Hospital de Clínicas de Porto Alegre, Universidade Federal do Rio Grande do Sul, Porto Alegre, Brazil.

Authors' contributions

JLN conceived the article, designed the spreadsheets, and drafted the manuscript. LBM and SCF revised the manuscript and approved the final version.

Competing interests

The authors declare that they have no competing interests.

Received: 4 August 2011 Accepted: 20 January 2012
Published: 20 January 2012

References

1. Stroup DF, Berlin JA, Morton SC, *et al*: **Meta-analysis of observational studies in epidemiology: a proposal for reporting. Meta-analysis Of Observational Studies in Epidemiology (MOOSE) group.** *JAMA* 2000, **283**:2008-12.
2. **Copenhagen: The Nordic Cochrane Centre, The Cochrane Collaboration, 2011.** *Review Manager (RevMan) [Computer program]. Version 5.1* [http://ims.cochrane.org/revman].
3. Rosenberg M: **MetaWin: Statistical Software for Meta-Analysis Version 2.** Sunderland, Massachusetts: Sinauer Associates; 2000 [http://www.metawinsoft.com].
4. Borenstein M, Hedges L, Higgins J, Rothstein H: **Comprehensive Meta-analysis Version 2.** Biostat, Englewood NJ; 2005 [http://www.meta-analysis.com].
5. Bax L, Yu L-M, Ikeda N, *et al*: **Development and validation of MIX: comprehensive free software for meta-analysis of causal research data.** *BMC Med Res Methodol* 2006, **6**:50.
6. Eddy DM: *FAST*PRO: Software for meta-analysis by the confidence profile method* Academic Press; 1992.
7. Wallace BC, Schmid CH, Lau J, *et al*: **Meta-Analyst: software for meta-analysis of binary, continuous and diagnostic data.** *BMC Med Res Methodol* 2009, **9**:80.
8. Borenstein M, Hedges LV, Higgins JPT, *et al*: *Introduction to Meta-Analysis.* 1 edition. Wiley; 2009.
9. Lipsey MW, Wilson D: *Practical Meta-Analysis.* 1 edition. Sage Publications, Inc; 2000.
10. Egger M, Smith GD, Altman D: *Systematic Reviews in Health Care: Meta-Analysis in Context.* 2 edition. BMJ Books; 2001.
11. Schriger DL, Altman DG, Vetter JA, *et al*: **Forest plots in reports of systematic reviews: a cross-sectional study reviewing current practice.** *Int J Epidemiol* 2010, **39**:421-9.
12. Higgins JPT, Thompson SG, Deeks JJ, *et al*: **Measuring inconsistency in meta-analyses.** *BMJ* 2003, **327**:557-60.

doi:10.1186/1756-0500-5-52
Cite this article as: Neyeloff *et al.*: Meta-analyses and Forest plots using a microsoft excel spreadsheet: step-by-step guide focusing on descriptive data analysis. *BMC Research Notes* 2012 5:52.

Submit your next manuscript to BioMed Central and take full advantage of:

- **Convenient online submission**
- **Thorough peer review**
- **No space constraints or color figure charges**
- **Immediate publication on acceptance**
- **Inclusion in PubMed, CAS, Scopus and Google Scholar**
- **Research which is freely available for redistribution**

Submit your manuscript at
www.biomedcentral.com/submit

BioMed Central
