# Supplementary material for: Trends in the prevalence of antenatal and postnatal depression in Bangladesh: A systematic review and meta-analysis
Source: Heliyon. 2025 Jan 14;11(2):e41955. doi: 10.1016/j.heliyon.2025.e41955 (PMC11787471; doi:10.1016/j.heliyon.2025.e41955)
Supplement: Multimedia component 5 [file mmc5.docx]

Supplementary Table: Search strategy of different databases.

| 1. PubMed | (("antenatal depression"[All Fields] OR "antepartum depression"[All Fields] OR "perinatal depression"[All Fields]) AND ("bangladesh"[MeSH Terms] OR "bangladesh"[All Fields] OR "bangladesh s"[All Fields])) AND ((humans[Filter]) AND (2000/1/1:2020/12/31[pdat]) AND (english[Filter]))  (("Postnatal depression"[All Fields] OR "postpartum depression"[All Fields] OR "perinatal depression"[All Fields]) AND ("bangladesh"[MeSH Terms] OR "bangladesh"[All Fields] OR "bangladesh s"[All Fields])) AND ((humans[Filter]) AND (2000/1/1:2020/12/31[pdat]) AND (english[Filter])) | 39 |
| --- | --- | --- |
| 1. Scopus | Antenatal depression AND postnatal depression in Bangladesh from 2000-2020 | 64 |
| 1. Cochrane | Antenatal depression AND Bangladesh (01/01/2000-31/12/2020)  Postnatal depression AND Bangladesh (01/01/2000-31/12/2020) | 57 |
| 1. BanglaJOL | Antenatal depression AND Bangladesh (01/01/2007-31/12/2020)  Postnatal depression AND Bangladesh (01/01/2007-31/12/2020)  Maternal depression AND Bangladesh 01/01/2007-31/12/2020) | 03 |
|  | Total screened articles | 163 |
