# Supplementary material for: Trends in the prevalence of antenatal and postnatal depression in Bangladesh: A systematic review and meta-analysis
Source: Heliyon. 2025 Jan 14;11(2):e41955. doi: 10.1016/j.heliyon.2025.e41955 (PMC11787471; doi:10.1016/j.heliyon.2025.e41955)
Supplement: Multimedia component 6 [file mmc6.docx]

| Author and ref | Doi | Study area | Study design | Sample size | Event | Prevalence | Scales | Questionnaire period | Risk factors/ associated factors | Quality |
| --- | --- | --- | --- | --- | --- | --- | --- | --- | --- | --- |
| (1)  H.E. Nasreen, 2010 | <https://doi.org/10.1186/1471-2458-10-515> | Mymensingh district  Random | Cohort study  Data:  2008-2009 | 720 | Antepartum depress: 132 | Antepartum depression: 18% | EPDS | Third trimester | Older age, Less education, lower body weight. | 1+1+1=3 |
| (2)  Gausia, K, 2009 | <https://doi.org/10.1007/s00737-009-0080-7> | Matlab, Dhaka  (Random) | Cohort study  Data:  2005 | 361 | Antenatal depression: 119 | Antepartum depression: 33% | EPDS-B | 34–35 weeks of pregnancy  (8-9 months)  Women 17-41 age | A history of being beaten by husband, having unhelpful or unsupportive mother in low, husband and family preference of male child. | 1+0+1=2 |
| (3)  Edhborg M, 2011 | <https://doi.org/10.1007/s00737-011-0221-7> | Rural sub-districts of Mymensingh district  Random | Cohort study  Data:  July 2007 to August 2008 | 720 | Depressive symptoms: 74 | Postnatal depress: 11% | EPDS | 2–3 months postpartum | Older, less educated, poorer, more intimate partner violence, lower emotional bonding with their infants due to girl. | 1+1+1=3 |
| (4)  Azad, 2019 | <https://doi.org/10.1371/journal.pone.0215735> | Urban slums (Gulshan, Mohakhali, Mohammadpur) from Dhaka city  Random | Cross sectional study  Data:  November-December 2017 | 376 | Depressive symptoms: 148 | Postnatal depress: 39.4% | EPDS | First 12 months following the childbirth. | Job involvement after child delivery, left job after pregnancy, unplanned pregnancy, perceived antenatal stress, at least one EPDS depressive symptom developed during pregnancy period, intimate partner violence before childbirth. | 1+0+1=2 |
| (5)  Kazi Nazira Sharmin,2019 | <https://doi.org/10.1016/j.midw.2019.03.014> | Dhaka city  (Population service and training center)  Purposively (Non-random) | Cohort study  Data: 2019 | 400  Age between 18 and 36 years | Depressive symptoms: 103 | Postnatal depress: 25.7% | EPDS | Followed up to 6–8 months  Up to 6 months category | Older age of mother, low family income, girl child and having more than one child. | 0+0+1=1 |
| (6)  Parveen akter, 2017 | <https://pubmed.ncbi.nlm.nih.gov/29208865/> | Dhaka  (BSMMU)  (Non-random) | Cross sectional study  Data:  July 2015 to December 2015 | 145 | Depressive symptoms: 18 | Postnatal depress: 12.4% | EPDS | 5-7 days following caesarean section | Maternal age, history of depression, lack of social support. | 0+0+1=1 |
| (7)  Maigun Edhborg, 2017 | [https://doi.org/10.1177/0886260517717489](https://doi.org/10.1177%2F0886260517717489) | Mymensingh district  (Random) | Data collected from a longitudinal study  Data:  July 2008- August 2009 | 656 | 208 | Postnatal depression 31.6% | EPDS | At 6 to 8 months postpartum  Up to 6 months category | Intimate partner violence including physical violence, emotional violence, sexual violence, mother’s perception of infant’s temperament (fussy-difficult, unadaptable, unpredictable) | 1+1+1=3 |
| (8)  Surkan, 2018 | <https://doi.org/10.1186/s12884-018-2097-2> | Gaibandha and Rangpur Districts (rural)  Random | Subset of Population based community trial and Randomized controlled trial.  Data:  August 2001 and October 2007. | Ante natal: 14,629  Postnatal: 31,422 | Ante natal depression: 1201  Postnatal  depression: 3965 | Ante natal depression: 8.2%  Postnatal depression:  12.6% | CES-D | third trimester  6 months postpartum  Up to 6 months category | Unwanted pregnancy either maternal, paternal or both, maternal age more than 30, poor SES, low maternal education was associated with depressive syndrome during prenatal and postnatal stage. | 1+1+1=3 |
| (9)  Gausia K, 2009 | <https://doi.org/10.1017/S0033291708004455> | Matlab, a rural subdistrict  (Random) | Cohort study  Data: 2008 | 346 | Postnatal  symptoms: 76 | Postnatal depression: 22% | EPDS-B | 6–8 weeks post-partum (1-2 months) | Post mental illness, depression in current pregnancy, perinatal death, poor relationship with mother-in-law, either the husband or the wife leaving home after a domestic quarrel. | 1+0+1=2 |
| (10)  Maureen M Black, 2009 | <https://doi.org/10.3945/ajcn.2008.26692E> | Matlab, Dhaka  (Random) | Subset of double  blinded RCT  Data:  2007 | 222 | Postnatal  symptoms: 114 | Postnatal depression: 52.7% (at 6 months)  52.1% at 12 months | CES-D | At 6 months and at 12 months | Poor infant growth (length-for-age) at 12 months, low maternal education level, poverty, low Home Inventory scores (home environment with limited stimulation), infant irritable temperament. | 1+0+1=2 |
| (11)  Zarina N. Kabir, 2014 | <https://doi.org/10.3402/gha.v7.24725> | Mymensingh district  (Random) | Cross-sectional  Data:  July 2008 to August 2009 | 660 | Postnatal  symptoms: 211 | Postnatal depression: 32% | EPDS | 6–8 months postpartum  Up to 6  months category | Poor relationship with husbands, low level of husband education, physical intimate partner violence after childbirth, perception of a fussy and difficult child. | 1+1+1=3 |
| (12)  J.D. Hamadani 2012 | <http://dx.doi.org/10.3329/jhpn.v30i2.11313> | Matlab, Dhaka | Cohort design  Data:  2007-2008 | 6 weeks :512  6months: 503 | Postnatal  Symptoms 6 weeks: 87  Postnatal  Symptoms 6 month: 55 | Postnatal depress: 6 weeks: 17%  Postnatal depress 6 months: 11% | EPDS | 6 weeks and  6 months postpartum | Not mentioned. | 1+1+1=3 |
| (13)  K Gausia, 2012 | <https://doi.org/10.3329/jhpn.v30i2.11310> | Matlab  Random | Cohort study  Data:  2007-2008 | 546 | Postnatal  Symptoms: 84 | Postnatal depression: 15.4% | EPDS | Six weeks of postpartum | Negative experience of childbirth significantly associated with PND. | 1+1+1=3 |
| (14)  Md. Jahirul Islam ,2017 | <https://doi.org/10.1371/journal.pone.0176211> | Chandpur district  (Random) | Cross-sectional  Data:  October 2015 and January 2016 | 426 | 150 | 35.2% | EPDS | Six months postpartum | Physical, sexual, and psychological IPV both during pregnancy and after childbirth were significantly associated with PPD | 1+0+1=2 |
| (15)  Nona M. Jiang 2017 | <https://doi.org/10.4269/ajtmh.17-0083> | Dhaka, Mirpur  Random | Cohort study  Data:  May 2011 to November 2014 | 78 weeks: 205  104 weeks: 422 | 78 weeks: 108  104 weeks: 200 | 78 weeks: 55.1%  104 weeks:48.2% | EPDS | 78 weeks and 104 weeks or  18 months and 24 months | Not mentioned. | 1+0+1=2 |
| (16)  Kaniz Gausia, 2011 | <https://doi.org/10.1186/1471-2458-11-451> | Matlab  Random | Perspective cohort study  Data:  2007- 2008 | 476 | 6 weeks postpartum: 113  6 months postpartum: 57 | 6 weeks postpartum 23.7%  6 months postpartum: 12% | EPDS-B | 6 weeks-6 months postpartum | Women with a perinatal death, worse relationships with husband, women who felt guilty about their results of last pregnancy outcome. | 1+0+1=2 |
| (17)  Kaniz Gausia, 2010 | <https://doi.org/10.3329/bmrcb.v36i1.1924> | Matlab, Dhaka  Random | Cohort study  Data: 2010 | 320 | Depressive symptoms: 64 | Postnatal depression: 20% | EPDS | 6-8 weeks postpartum  (1-2 months) | Diarrhea, malnutrition among infants. | 1+0+1=2 |
| (18)  Sheikh Jamal Hossain, **2020** | <https://doi.org/10.3390/ijerph17134727> | Ullapara sub district of Rural Bangladesh  (Random) | Cross sectional  Data: 2017 | 591 | 307 | Postnatal depression 51.7% | SRQ-20  Self-Reporting Questionnaire (SRQ-20) | Age: 16–45 years  6–16 months postpartum | Older mother, low level of parental education, no family food security, Violence against woman | 1+1+0=2 |

Analysis

1. Ante partum 2. Postpartum

For-Subset analysis.

1. Data collected up to 2010 vs after 2010, I have added data collection time in the study design column.
2. Quality assessment (high vs low)

1. In postpartum-

- **Immediate to 6-8 weeks**
- **Measured at 6-8 months**
- **More than 6-8 months**

For quality assessment:

If Random give 1

If use EPDS or CESD give 1, for self- Reporting questionnaire 0

If the sample size is less than 500 give 0, more than 500 give 1

*** Low Home Inventory scores:  Infants are not receiving either the nutrients or caregiving that they need to grow and develop

**SCALES:**

- Edinburgh Postnatal Depression Scale = EPDS
- Self-Reporting Questionnaire = SFQ
- The Center for Epidemiologic Studies Depression Scale = CES-D

1. Nasreen HE, Kabir ZN, Forsell Y, Edhborg M. Low birth weight in offspring of women with depressive and anxiety symptoms during pregnancy: results from a population based study in Bangladesh. BMC Public Health. 2010;10(1):515.

2. Gausia K, Fisher C, Ali M, Oosthuizen J. Antenatal depression and suicidal ideation among rural Bangladeshi women: a community-based study. Archives of Women's Mental Health. 2009;12(5):351.

3. Edhborg M, Nasreen H-E, Kabir ZN. Impact of postpartum depressive and anxiety symptoms on mothers’ emotional tie to their infants 2–3 months postpartum: a population-based study from rural Bangladesh. Archives of Women's Mental Health. 2011;14(4):307.

4. Azad R, Fahmi R, Shrestha S, Joshi H, Hasan M, Khan ANS, et al. Prevalence and risk factors of postpartum depression within one year after birth in urban slums of Dhaka, Bangladesh. PLOS ONE. 2019;14(5):e0215735.

5. Sharmin KN, Sarwar N, Mumu SJ, Taleb DRA, Flora MS. Postnatal depression and infant growth in an urban area of Bangladesh. Midwifery. 2019;74:57-67.

6. Parveen T, Akhter S, Parveen HH, Noor F, Kazal RK. Assessment of Postnatal Depression by Edinburgh Postnatal Depression Scale in a Tertiary Care Hospital of Bangladesh. Mymensingh medical journal : MMJ. 2017;26(4):783-9.

7. Edhborg M, E-Nasreen H, Kabir ZN. Impact of Intimate Partner Violence on Infant Temperament. 2020;35(21-22):4779-95.

8. Surkan PJ, Strobino DM, Mehra S, Shamim AA, Rashid M, Wu LS-F, et al. Unintended pregnancy is a risk factor for depressive symptoms among socio-economically disadvantaged women in rural Bangladesh. BMC Pregnancy and Childbirth. 2018;18(1):490.

9. Gausia K, Fisher C, Ali M, Oosthuizen J. Magnitude and contributory factors of postnatal depression: a community-based cohort study from a rural subdistrict of Bangladesh. Psychological Medicine. 2009;39(6):999-1007.

10. Black MM, Baqui AH, Zaman K, Arifeen SE, Black RE. Maternal depressive symptoms and infant growth in rural Bangladesh. The American Journal of Clinical Nutrition. 2009;89(3):951S-7S.

11. Kabir ZN, Nasreen H-E, Edhborg M. Intimate partner violence and its association with maternal depressive symptoms 6–8 months after childbirth in rural Bangladesh. Global Health Action. 2014;7(1):24725.

12. Hamadani JD, Tofail F, Hilaly A, Mehrin F, Shiraji S, Banu S, et al. Association of postpartum maternal morbidities with children's mental, psychomotor and language development in rural Bangladesh. Journal of health, population, and nutrition. 2012;30(2):193-204.

13. Gausia K, Ryder D, Ali M, Fisher C, Moran A, Koblinsky M. Obstetric complications and psychological well-being: experiences of Bangladeshi women during pregnancy and childbirth. Journal of health, population, and nutrition. 2012;30(2):172-80.

14. Islam MJ, Broidy L, Baird K, Mazerolle P. Intimate partner violence around the time of pregnancy and postpartum depression: The experience of women of Bangladesh. PLOS ONE. 2017;12(5):e0176211.

15. Jiang NM, Tofail F, Ma JZ, Haque R, Kirkpatrick B, Nelson CA, et al. Early Life Inflammation and Neurodevelopmental Outcome in Bangladeshi Infants Growing Up in Adversity. 2017;97(3):974-9.

16. Gausia K, Moran AC, Ali M, Ryder D, Fisher C, Koblinsky M. Psychological and social consequences among mothers suffering from perinatal loss: perspective from a low income country. BMC Public Health. 2011;11(1):451.

17. Gausia K, Ali M, Ryder D. Diarrhea in Bangladeshi infants and its association with postnatal depression. Bangladesh Med Res Counc Bull. 2010;36(1):32-4.

18. Hossain SJ, Roy BR, Hossain AT, Mehrin F, Tipu SMMU, Tofail F, et al. Prevalence of Maternal Postpartum Depression, Health-Seeking Behavior and Out of Pocket Payment for Physical Illness and Cost Coping Mechanism of the Poor Families in Bangladesh: A Rural Community-Based Study. Int J Environ Res Public Health. 2020;17(13):4727.
