# Supplementary material for: Trends in the prevalence of antenatal and postnatal depression in Bangladesh: A systematic review and meta-analysis
Source: Heliyon. 2025 Jan 14;11(2):e41955. doi: 10.1016/j.heliyon.2025.e41955 (PMC11787471; doi:10.1016/j.heliyon.2025.e41955)
Supplement: Multimedia component 7 [file mmc7.docx]

## **Table 1: AMSTAR-2 checklist for Systematic Reviews**

| **Domain** | **Response Options** |
| --- | --- |
| **1. Did the research questions and inclusion criteria for the review include the components of PICO?** | **Yes/No/Partial Yes** |
| **2. Was the review protocol registered before the conduct of the review (e.g., in PROSPERO)?** | **Yes/No** |
| **3. Did the review authors explain their selection of study designs for inclusion?** | **Yes/No** |
| **4. Did the review authors use a comprehensive literature search strategy?** | **Yes/No/Partial Yes** |
| **5. Did the review authors perform study selection in duplicate?** | **Yes/No** |
| **6. Did the review authors perform data extraction in duplicate?** | **Yes/No** |
| **7. Did the review authors provide a list of excluded studies and justify the exclusions?** | **Yes/No/Partial Yes** |
| **8. Did the review authors describe the included studies in adequate detail?** | **Yes/No/Partial Yes** |
| **9. Did the review authors use a satisfactory technique for assessing the risk of bias (RoB) in individual studies?** | **Yes/No/Partial Yes** |
| **10. Did the review authors report on the sources of funding for the studies included in the review?** | **Yes/No** |
| **11. If meta-analysis was performed, did the review authors use appropriate methods for statistical combination of results?** | **Yes/No/Not Applicable** |
| **12. If meta-analysis was performed, did the review authors assess the potential impact of risk of bias in individual studies on the results of the meta-analysis or other evidence synthesis?** | **Yes/No/Not Applicable** |
| **13. Did the review authors account for RoB in primary studies when interpreting/discussing the results of the review?** | **Yes/No** |
| **14. Did the review authors provide a satisfactory explanation for, and discussion of, any heterogeneity observed in the results of the review?** | **Yes/No/Not Applicable** |
| **15. If they performed quantitative synthesis, did the review authors carry out an adequate investigation of publication bias (small study bias) and discuss its likely impact on the results?** | **Yes/No/Not Applicable** |
| **16. Did the review authors report any potential sources of conflict of interest, including any funding they received for conducting the review?** | **Yes/No** |
